# Tumor-Induced Rewiring of Splenic Niches: from Immune Organ to Cancer Accomplice

**DOI:** 10.7150/ijbs.127169

**Published:** 2026-02-04

**Authors:** Tong Yuan, Junjie Liu, Chunyu Zhang, Xing Lv, Guan Tan, Lin Xue, Erlei Zhang, Huifang Liang, Zhiyong Huang

**Affiliations:** 1 Division of Hepato-Pancreato-Biliary Surgery, Tongji Hospital, Tongji Medical College, Huazhong University of Science and Technology, Wuhan, 430030, China; 2 Department of Urology, Tongji Hospital, Tongji Medical College, Huazhong University of Science and Technology, Wuhan, 430030, China.

**Keywords:** tumor, splenic niches, extramedullary hematopoiesis, neuroimmune crosstalk, therapeutic strategies

## Abstract

The spleen is the largest secondary lymphoid organ in humans. Beyond its classical role in clearance of senescent erythrocytes, it functions as a pivotal node in systemic immune surveillance. Emerging evidence indicates that tumor can remotely remodel splenic niches through a spectrum of soluble mediators, thereby accelerating tumor initiation and progression. Tumor-derived signals divert splenic hematopoietic stem and progenitor cells (HSPCs) toward myeloid- and erythroid-biased extramedullary hematopoiesis (EMH), expanding myeloid-derived suppressor cells (MDSCs) and erythroid progenitor cells (EPCs) that collectively foster immune evasion and metastatic cascades. Consequently, splenic resident immune cells, stromal cells and EMH-related pathways have surfaced as actionable therapeutic targets. In parallel, bidirectional crosstalk between the autonomic nervous system and splenic immunity fine-tunes homeostasis, systemic inflammation and antitumor responses—fueling rising interest in splenic neuromodulation as a therapeutic strategy. In addition, spleen-targeted nanoplatforms are emerging as promising tools to deliver immunomodulatory payloads with improved precision. Nonetheless, inherent structural and functional disparities between human and murine spleens complicate clinical translation of pre-clinical findings. This review provides a concise overview of human lymphoid organs and their functions, with a particular focus on splenic anatomy, cellular composition, and neural regulation. It further delineates tumor-induced splenic rewiring and discusses the prospects of exploiting the spleen as both a biomarker and a therapeutic target in oncology.

## 1. Introduction

In both humans and mice, the spleen represents the most prominent secondary lymphoid organ, playing essential roles in filtering circulating blood, clearing aged red blood cells, and initiating immune defenses against pathogens present in the bloodstream^1^. Accelerated progress in tumor immunology has reframed the spleen from a passive participant to an active regulator of cancer progression. Although overt splenic metastasis is rare, tumor-derived cytokines, chemokines and extracellular vesicles convert the spleen into a “central platform” for immune tolerance and hematopoietic reprogramming^2^. In breast cancer^3^, melanoma^4^, hepatocellular carcinoma (HCC)^5^ and other tumor models, hematopoietic stem and progenitor cells (HSPCs) are redirected toward myeloid- and erythroid-biased extramedullary hematopoiesis (EMH). This expands myeloid-derived suppressor cells (MDSCs) and erythroid progenitor cells (EPCs) which in turn suppress cytotoxic T-cell activity and facilitate metastatic spread^2^, ^6^. Hence, splenic immune and stromal compartments, together with EMH-driving signals, represent promising therapeutic entry points. A further layer of complexity arises from the interaction between the nervous system and splenic immunity^7^. Sympathetic nerve endings encircle central arterioles (CAs) in the white pulp (WP) and release various neurotransmitters, thereby directly modulating T cells, B cells and macrophages under both steady-state and pathological conditions^8^, ^9^. Vagal inputs can modulate sympathetic outflow via the coeliac ganglion, thereby indirectly regulating neuroimmune interactions within the spleen^10^. Parallel clinical studies have linked increases in splenic volume and ¹⁸F-FDG uptake with poor prognosis in several malignancies, underlining the potential of splenic morpho-metabolic changes as non-invasive biomarkers^11^.

Against this backdrop, this review provides a concise overview of human lymphoid organs and their functions, and then outlines splenic anatomical compartments, cellular constituents and innervation. It further explains how tumors rewire the splenic niches, evaluates multiple spleen-targeted therapeutic strategies and discusses the clinical potential of splenic morpho-metabolic characteristics as non-invasive biomarkers.

## 2. Division and Cooperation of lymphoid Organs

**Figure 1:**
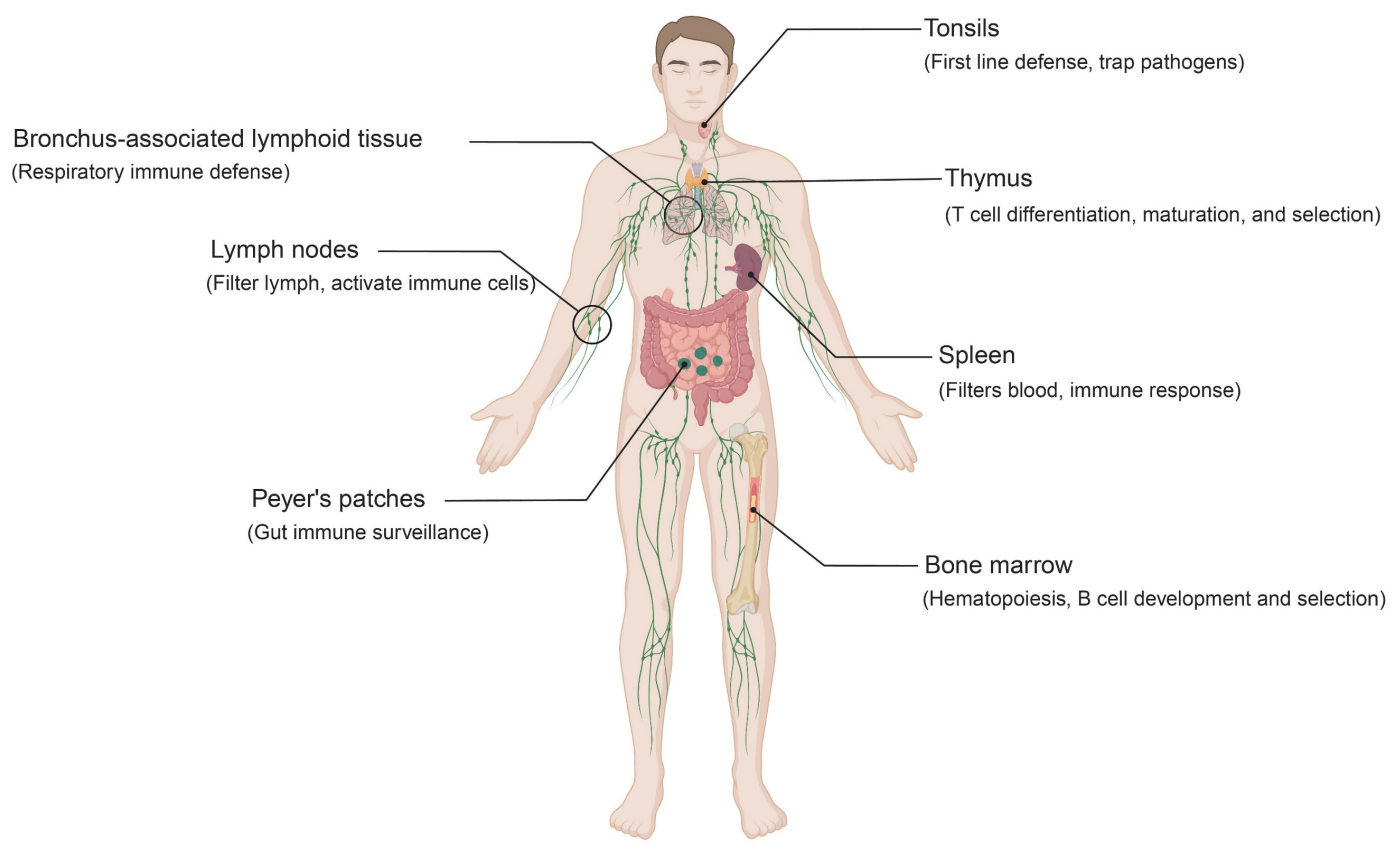
** Human lymphoid organs and their functions.** This schematic illustrates the primary and secondary lymphoid organs involved in immune regulation. The bone marrow supports hematopoiesis as well as B cell development and selection. The thymus governs T cell differentiation, maturation, and selection. Secondary lymphoid organs include the spleen, lymph nodes, and mucosa-associated lymphoid tissue (MALT). Together, they coordinate immune surveillance and orchestrate immune responses. Specifically, the spleen filters blood and coordinates systemic immunity, while lymph nodes filter lymph and promote immune cell activation. MALT comprises structures such as the tonsils, Peyer's patches, and bronchus-associated lymphoid tissue (BALT). Tonsils provide a first line of defense at mucosal surfaces, while Peyer's patches and BALT enable local immune surveillance in the gut and respiratory tract. **Abbreviations:** MALT, mucosa-associated lymphoid tissue; BALT, bronchus-associated lymphoid tissue.

The immune system is a hierarchically organized defense network responsible for protecting the host against invading pathogens and monitoring for malignant transformation. Traditionally, it is divided into innate and adaptive immunity^12^. Innate immunity relies on host-encoded mechanisms such as physical barriers, soluble factors, and innate immune cells including neutrophils, dendritic cells (DCs), and natural killer (NK) cells^13^. It is characterized by rapid, broad, and non-specific responses, primarily functioning in the early phase of pathogen invasion to recognize and eliminate threats while providing signals that bridge to adaptive immunity. In contrast, adaptive immunity depends on B cells and T cells, which mediate highly specific antigen recognition and establish durable immunological memory^14^. Importantly, effective immune responses are contingent on the specialized structures and microenvironments of primary and secondary lymphoid organs, which together form the anatomical framework of the immune system (Figure 1).

Primary lymphoid organs consist of the bone marrow and thymus (Figure 1). In the bone marrow, HSPCs differentiate into lymphoid and myeloid lineages^15^. B cells complete their development and selection within this niche, whereas T cell precursors arise in the bone marrow and subsequently migrate to the thymus^16^. Antigen-presenting cells (APCs), such as macrophages and DCs, are also generated in the bone marrow and contribute to early immune recognition of foreign molecules in the blood and tissues^17^, ^18^. The thymus is the primary site for T cell differentiation, maturation, and selection. Immature T cells undergo positive selection, which ensures the functional expression of the T cell receptor (TCR), and negative selection, which eliminates autoreactive clones^19^. This dual process yields a repertoire of mature T cells with appropriate antigen specificity and affinity while preserving self-tolerance^20^, ^21^.

Secondary lymphoid organs include mucosa-associated lymphoid tissue (MALT), lymph nodes, and the spleen (Figure 1)^22^, ^23^. Acting as a strategically distributed “filtering network”, they monitor and process extracellular fluids, including interstitial fluid, lymph, and blood. This supports targeted immune responses against potential pathogens. MALT, distributed along the respiratory and gastrointestinal tracts, comprises structures such as the tonsils, Peyer's patches, and bronchus-associated lymphoid tissue (BALT)^24^. Among these, the tonsils represent a central component of mucosal immunity^25^. Within their specialized crypt architecture, B cells undergo antigen-driven differentiation into plasma cells that secrete IgA. These plasma cells migrate to mucosal surfaces, where secretory IgA provides antigen-specific protection against respiratory and gastrointestinal pathogens^26^, ^27^. Similarly, Peyer's patches function as key sites of gut immune surveillance, while BALT plays a central role in respiratory immune defense^28^, ^29^. Lymph nodes, widely distributed along the lymphatic system, serve as key immune surveillance hubs. Through highly organized compartmentalization, they coordinate immune responses: antigens and professional APCs are delivered via afferent lymphatics, initiating T cell responses in the paracortical zone, while follicular B cells in the cortex undergo activation and germinal center reactions^30^. Specialized high endothelial venules (HEVs) within lymph nodes promote lymphocyte recirculation, thereby sustaining systemic immune cell homeostasis and ensuring the rapid deployment of effector cells throughout the body^31^. The spleen, the largest secondary lymphoid organ, occupies a central role in systemic immunity^1^. It not only clears senescent red blood cells and participates in innate defense but also supports adaptive responses against blood-borne pathogens through T and B cell activation.

Although anatomically dispersed, lymphoid organs are functionally integrated. Lymphocytes generated in the bone marrow and thymus migrate to secondary organs, where, with the assistance of APCs, they are activated and subsequently execute immune responses in circulation and peripheral tissues^32^. Adaptive immunity, with its long-lasting specificity, complements the rapid but transient defense of innate immunity: the former ensures durable protection, while the latter provides immediate pathogen clearance and primes adaptive responses^12^. Furthermore, the specialized structures and cellular ecosystems of lymphoid organs dictate immune cell development, activation, and memory formation. Disruption of these processes predisposes to infections or autoimmune disorders and simultaneously creates opportunities for tumors to reshape immune homeostasis. In recent years, growing attention has been directed toward the interaction between the spleen and tumors^6^, ^33^. In the following section, we will provide a detailed discussion of the normal physiological structure and functions of the spleen, as well as its rewiring in the tumor context.

## 3. Anatomical Architecture, Cellular Constituents and Neural Innervation of the Spleen

### 3.1 Structural Compartments

**Figure 2:**
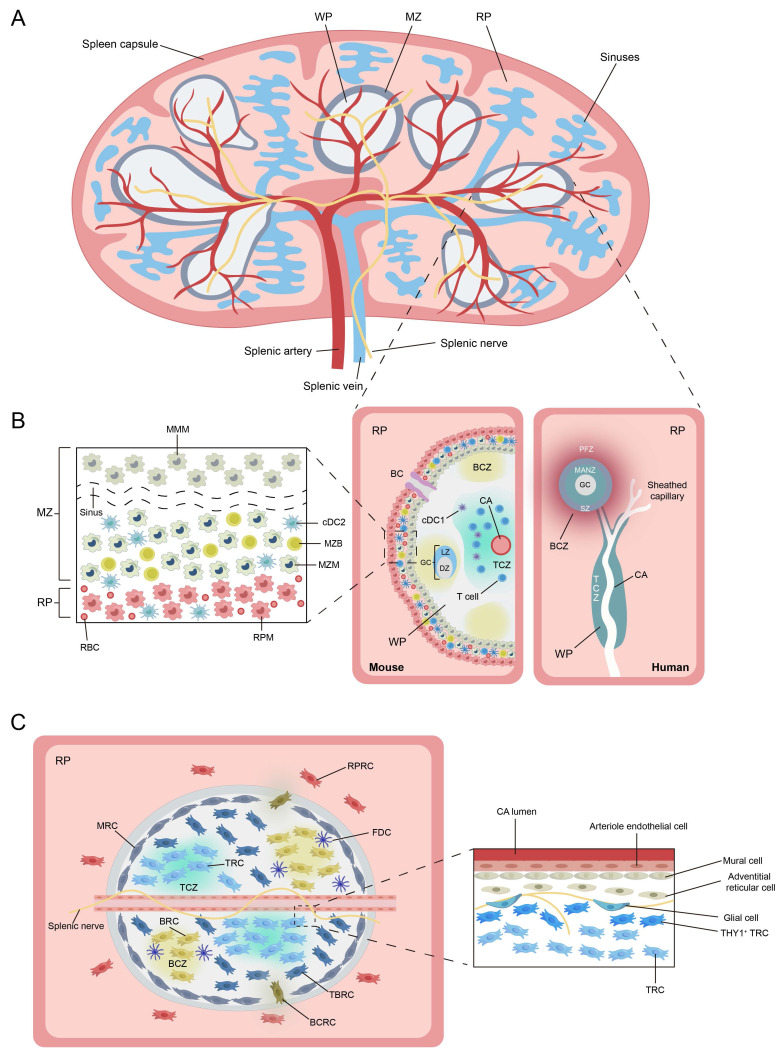
** Schematic representation of the splenic structure and its immune and stromal cell composition. (A)** Overview of the anatomical structure of the mouse spleen. **(B)** Immune cellular architecture of the spleen in steady state in mouse (left) and human (right). Notable differences exist between species, especially in the organization of the T cell zone (TCZ) and B cell zone (BCZ) within the white pulp (WP), and in the boundary between WP and red pulp (RP). In mice, this boundary is defined by the marginal zone (MZ), whereas in humans, it corresponds to the perifollicular zone (PFZ). The PFZ is further subdivided into the mantle zone (MANZ), superficial zone (SZ), and the outer PFZ layer. In mice, the layered distribution of macrophage subsets in the MZ has been well characterized (left panel), with CD169⁺ marginal metallophilic macrophages (MMMs) forming a concentric ring around the WP, alongside marginal zone macrophages (MZMs) and marginal zone B cells (MZBs). In humans, such organization is less defined; however, MZB cells are known to surround activated B cells, forming a germinal center (GC) and a surrounding corona. The localization of dendritic cell (DC) subsets in the mouse spleen is also depicted: Conventional type 1 DCs (cDC1s) are primarily located in the TCZ, while conventional type 2 DCs (cDC2s) are mainly distributed in the RP and MZ. A subset of SIRPα⁺cDC2s also localizes to the bridging channels (BCs). **(C)** Stromal cell niches of the spleen. In the WP, T cell zone reticular cells (TRCs) attract and retain T cells and cDC1s. T-B border reticular cells (TBRCs) demarcate the interface between TCZ and BCZ. Within BCZ, CXCL13⁺ B cell zone reticular cells (BRCs) support compartmental structure and include follicular dendritic cells (FDCs). In the MZ, marginal reticular cells (MRCs) are associated with MMMs and maintain local architecture. Specialized red pulp reticular cells (RPRCs) in the RP provide structural support and survival cues to macrophages and plasma cells within the cords. The BC is formed by bridging channel reticular cells (BCRCs), providing a distinct microenvironment for SIRPα⁺ cDC2s. The right inset shows the perivascular stromal niche in the WP. Mural cells line the wall of the central arterioles (CAs), surrounded by CD34⁺ adventitial reticular cells, which may serve as progenitors for fibroblastic reticular cells. The splenic nerve runs along the CAs and extends into the TCZ, where it is supported by podoplanin-expressing glial cells. A population of THY1⁺ TRCs, functionally distinct from conventional TRCs, resides exclusively in this perivascular niche and is possibly derived from CD34⁺ adventitial reticular cells. These THY1⁺ TRCs, together with CXCL9⁺ TRCs, contribute to the architecture of the TCZ. **Abbreviations:** WP, white pulp; RP, red pulp; MZ, marginal zone; PFZ, perifollicular zone; MANZ, mantle zone; SZ, superficial zone; TCZ, T cell zone; BCZ, B cell zone; GC, germinal center; CA, central arteriole; BC, bridging channel; DC, dendritic cell; cDC1, conventional type 1 dendritic cell; cDC2, conventional type 2 dendritic cell; MMM, marginal metallophilic macrophage; MZM, marginal zone macrophage; MZB, marginal zone B cell; RPM, red pulp macrophage; TRC, T cell zone reticular cell; TBRC, T-B border reticular cell; BRC, B cell zone reticular cell; FDC, follicular dendritic cell; MRC, marginal reticular cell; RPRC, red pulp reticular cell; BCRC, bridging channel reticular cell; SIRPα, signal regulatory protein alpha; CXCL, C-X-C motif chemokine ligand; CD, cluster of differentiation; THY1, thymocyte differentiation antigen 1.

Current descriptions of the different structural compartments of the spleen are based almost entirely on studies of mouse or other rodent spleens. Macroscopically, the spleen is enclosed by a thick connective tissue capsule, from which trabeculae extend inward to anchor vascular structures and subdivide the organ into distinct lobular units (Figure 2A)^34^. Functionally, splenic tissue is organized into WP and red pulp (RP). In mice, the WP occupies a substantial proportion of the spleen and is separated from the RP by a well-defined marginal zone (MZ) (Figure 2B)^34^. The WP orchestrates adaptive immune responses, whereas the RP filters ageing or damaged blood cells and surveys blood-borne pathogens^35^. In contrast to lymph nodes, the spleen does not possess afferent lymphatic vessels; instead, all antigens and cells enter via the bloodstream^36^. Whether an efferent lymphatic pathway exists in the WP remains debated^37^.

#### 3.1.1 White Pulp

In mice, the WP is composed of lymphoid follicles arranged around CAs (Figure 2B). Each CA is surrounded by a MZ, which gradually transitions into the RP^35^. Within the WP, the T cell zone (TCZ) and B cell zone (BCZ) are spatially segregated. The TCZ, also termed the periarteriolar lymphoid sheath (PALS), surrounds CAs and harbors naive and activated T cells. The TCZ is the primary site of T cell activation, where DCs present antigen and initiate cellular immune responses. Chemokine receptor CCR7 and its ligands CCL19 and CCL21 are essential for maintaining T-cell confinement; their absence scatters T cells throughout the organ^34^, ^38^, ^39^. Adjacent to the TCZ lies the BCZ, comprising lymphoid follicles that house the key cells required for B cell activation and survival^40^. The BCZ is where germinal centers (GCs) form to generate humoral immunity and produce antibodies. The BCZ also contains follicular dendritic cells (FDCs) that present native antigen and secrete CXCL13 to sustain follicular architecture (Figure 2C)^41^.

#### 3.1.2 Red Pulp

**Figure 3:**
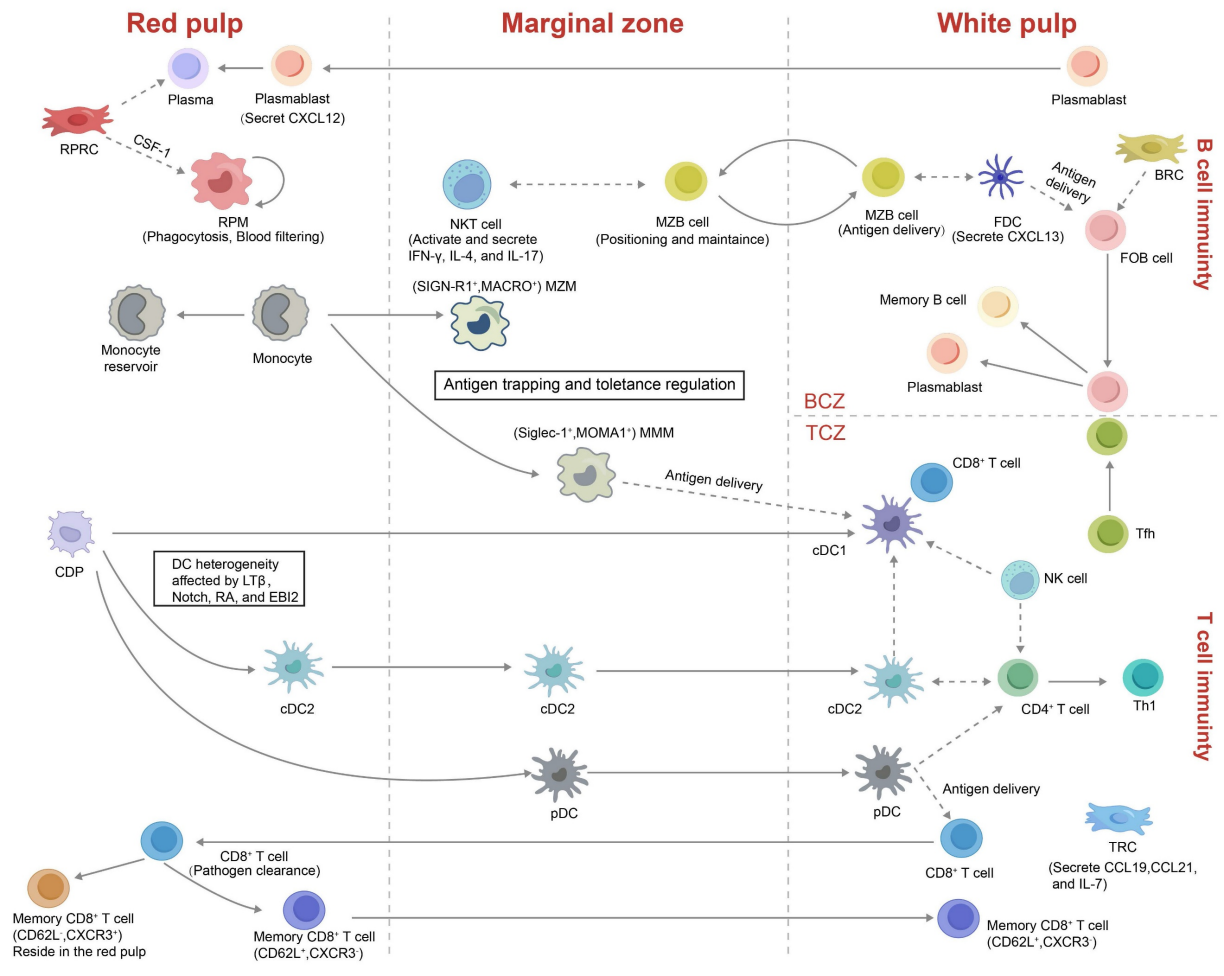
** Localization and functional illustration of splenic immune and stromal cells.** This schematic provides an overview of various innate, adaptive immune, and stromal cell types within the spleen, highlighting their distinct roles under pathological conditions. The illustration also depicts the origins, spatial localization, migratory behavior, and cellular interactions of these subsets across different splenic compartments. **Abbreviations:** CDP, common dendritic cell progenitor; FDC, follicular dendritic cell; FOB, follicular B cell; LTβ, lymphotoxin beta; RA, retinoic acid; EBI2, Epstein-Barr virus-induced G protein-coupled receptor 2; BCZ, B cell zone; TCZ, T cell zone; MZM, marginal zone macrophage; MMM, marginal metallophilic macrophage; MZB cell, marginal zone B cell; FRC, fibroblastic reticular cell; TRC, T zone reticular cell; Tfh, T follicular helper cell; RPM, red pulp macrophage; RPRC, red pulp reticular cell; CSF-1, colony-stimulating factor-1.

In mice, the RP consists of a reticular-fiber framework supporting lymphocytes, neutrophils, mast cells and abundant macrophages^1^. These cells are distributed throughout the splenic cords, which envelop the expansive venous sinusoids. Although WP is the primary site for launching adaptive immune responses, the RP plays a central role in executing effector functions^42^. Under the influence of the chemokine CXCL12, plasmablasts relocate from the WP to the RP, where they secrete antibodies into the circulation (Figure 3)^43^. Similarly, activated CD8⁺ T cells are recruited to the RP to confront invading antigens^44^. Moreover, the RP serves as a site of EMH and acts as a reservoir for circulating monocytes, platelets, and erythrocytes^37^.

#### 3.1.3 Marginal Zone

The murine MZ is a circumferential layer rich in B cells, macrophages and DCs (Figure 2B)^45^. Marginal zone B cells (MZBs), which possess innate-like features, are anchored via integrins LFA-1 and α4β7 (binding ICAM-1 and VCAM-1) and retained by sphingosine-1-phosphate (S1P) signaling^46^. Two specialized macrophage subsets reside here: marginal metallophilic macrophages (MMMs), expressing CD169/Siglec-1 and MOMA-1, and marginal-zone macrophages (MZMs), expressing MARCO and SIGN-R1^34^. Both subsets capture and process blood-borne antigens via pattern-recognition receptors^34^. Bridging channels (BCs) are a series of small conduits located between the MZ and RP of the spleen, formed at gaps between MMMs and MZMs surrounding the CAs^1^. Within BCs, specialized reticular cells secrete the chemokine CCL21, which attracts both naive and activated lymphocytes and guides their transit from the MZ into the RP, thereby enhancing surveillance of blood-borne antigens^44^.

#### 3.1.4 Species-Specific Differences in Human and Murine Splenic Architecture

Although the functions of immune cells and their regional niches in the spleen are largely similar between mice and humans, fundamental differences exist in overall architecture and in certain cell populations^40^. In humans, the RP occupies most of the splenic volume, in contrast to mice. Within the human WP, the BCZ is the predominant compartment, whereas the TCZ is relatively reduced. The TCZ and BCZ in humans are arranged in a “grape-on-a-vine” pattern, with the CA traversing both zones^1^. This differs from mice, where concentric rings of BCZ surround a central TCZ. The circulatory pattern also differs between species^34^. In mice, the RP operates a mixed open-closed circulation. In humans, the RP is generally considered to function as a fully open system. Blood leaves the capillaries, enters the splenic cords, and only then re-enters the venous sinuses, thereby maximizing contact with phagocytes^34^.

The most striking difference between murine and human spleens lies at the interface between WP and RP^1^. The human spleen lacks a classical MZ. Instead, a perifollicular zone (PFZ) forms the main boundary between WP and RP^35^. This region in humans is characterized by sheathed capillaries rather than the marginal sinus described in rodents. It also lacks MMMs and phenotypically well-defined MZMs, although macrophages are still present around the follicular periphery. Within the PFZ, erythrocytes, granulocytes, and monocytes are intermingled with additional MAdCAM-1⁺ stromal cells^47^. BCs are morphologically distinct structures in the murine spleen but have not yet been demonstrated in humans. Functionally, in mice, DCs together with MZBs capture and transport blood-borne antigens into the WP, supporting T- and B-cell surveillance of circulating antigens^38^. In humans, by contrast, antigen uptake and presentation rely mainly on DCs that migrate from the PFZ into the WP.

Whether these differences in organization alter the initiation or execution of immune responses in the mouse versus human spleen remains unclear. Applying advanced imaging and high-dimensional phenotypic profiling to human spleen tissue will be essential to clarify structural and functional parallels and discrepancies in the marginal region between species. Such insights will allow a more accurate assessment of how far murine immunology can be extrapolated to human biology and disease.

### 3.2 Cellular Constituents

#### 3.2.1 Resident Lymphoid Cells

T and B lymphocytes dominate splenic immunity yet occupy distinct niches orchestrated by stromal networks, integrins and chemokines^41^, ^48^. Naive CD4⁺ T cells predominantly reside at the periphery of the PALS, whereas CD8⁺ T cells are mainly localized to its central region^38^. Activated T-follicular helper (Tfh) cells up-regulate CXCR5 to move toward CXCL13-rich BCZ, whereas antigen-experienced B cells up-regulate CCR7 to approach the T-B border, enabling cognate interaction and GCs formation (Figure 3)^49^, ^50^. Activated CD8⁺ effector T cells traverse BCs into the MZ and RP to eliminate pathogens^44^. Some memory CD8⁺ T cells (CD62L⁺CXCR3⁻) return to the PALS, whereas CD62L⁻CXCR3⁺ memory CD8⁺ T cells are retained in the RP^51^, ^52^.

The spleen contains multiple B cell subsets, chiefly follicular B cells (FOBs) and MZBs. FOBs are located within follicles in the WP and mediate T cell-dependent adaptive immune responses (Figure 3)^53^. Upon antigen recognition, FOBs relocate to the T-B border, where they engage with Tfh cells and subsequently enter GCs. There, they undergo somatic hypermutation, refine antigen affinity, and switch immunoglobulin isotypes, eventually giving rise to plasmablasts or long-lived memory B cells^54^. By contrast, MZBs reside at the interface between the MZ and RP, where they rapidly capture blood-borne antigens and participate in both T cell-independent and -dependent immune responses^55^. MZBs recognize immune complexes via complement receptors (e.g., CR1/CR2) and, guided by sphingosine-1-phosphate (S1P) gradients, shuttle dynamically between the MZ and RP, thereby enhancing antigen delivery and antibody production^56^, ^57^. Regulatory B cells (Bregs), which have garnered increasing attention in recent years, predominantly arise from MZB and FOB populations and localize at the MZ-WP interface. Through the release of immunoregulatory cytokines, including interleukin-10 (IL-10) and transforming growth factor-beta (TGF-β), these cells influence T cell activity, restrain inflammation, and prevent autoimmunity^58^-^60^.

NK cells reside mainly in the RP at steady state^61^. Upon immune challenge they migrate into the WP, secrete interferon-γ to bias CD4⁺ T cells toward a Th1 phenotype and promote DC maturation to enhance innate immunity (Figure 3)^62^. In parallel, natural killer T (NKT) cells are enriched in both the MZ and RP, where they engage with MZBs to detect blood-borne antigens and enhance T cell priming^63^. The precise localization of these lymphocyte subsets within the splenic microenvironment is essential for their immune functions and antigen sensing.

#### 3.2.2 Resident Myeloid Cells

Splenic myeloid populations include macrophages, DCs and monocytes (Figure 3). The two specialised macrophage subsets, MMMs and MZMs, derive from bone-marrow progenitors and require macrophage colony-stimulating factor (M-CSF) and liver X receptor-α (LXRα) for their development^64^. MZMs engage MZBs in early antibacterial defense, whereas MMM pseudopods extend across the marginal sinus into the WP to cooperate with DCs in antigen presentation^37^, ^65^. Both subsets support peripheral tolerance and can be replenished rapidly from monocytes during inflammation^66^. Another subset of macrophages is the red pulp macrophages (RPMs), which reside in the RP and are specialized in phagocytosing senescent erythrocytes and blood-borne pathogens, thereby playing a vital role in iron recycling and systemic blood filtration^67^, ^68^.

Splenic DCs constitute about 3% of total CD45⁺ spleen cells and comprise conventional type 1 DCs (cDC1s), conventional type 2 DCs (cDC2) and plasmacytoid DCs (pDCs). cDC1s (CD8α⁺CD11b⁻) are primarily positioned within the TCZ, where they specialize in cross-presenting apoptotic antigens to CD8⁺ T cells (Figure 2B, Figure 3)^69^. In contrast, cDC2s (CD8α⁻CD11b⁺) reside mainly in the RP and MZ, where they present MHC class II-restricted peptides to CD4⁺ T cells. A subset of SIRPα⁺ cDC2s also localizes to the BCs, where they are retained by stromal-derived oxysterols. These cells are essential for initiating CD4⁺ T-cell responses^70^, ^71^. pDCs, which arise from both common dendritic cell precursors (CDPs) and IL-7 receptor-positive lymphoid progenitors, are potent producers of type I interferons, IL-12, and IL-18 upon activation. These cytokines enhance NKT and CD8⁺ T cell activity and promote the differentiation of CD4⁺ T cells toward a Th1 phenotype^72^.

Splenic monocytes are recruited from the peripheral blood to the spleen to replenish the macrophage pool and, under chemokine cues, migrate to the MZ to support T cell-independent MZBs responses^73^. At steady state, undifferentiated CX3CR1^int^Ly6C^hi^ and CX3CR1^hi^Ly6C^lo^ monocytes aggregate in subcapsular regions of the RP, forming a reservoir that can differentiate into macrophages or atypical DCs^74^. During inflammation or tissue injury, these stored monocytes can swiftly exit to peripheral sites, supplementing bone marrow-derived monocytes^37^.

#### 3.2.3 Stromal Cells

The splenic microenvironment consists of a variety of heterogeneous stromal cells, primarily including endothelial cells, mural cells, and fibroblastic reticular cells (FRCs). Among FRCs, several subtypes have been identified, such as T zone reticular cells (TRCs), B zone reticular cells (BRCs), red pulp reticular cells (RPRCs), and adventitial reticular cells (Figure 2C)^75^.

The WP is a highly organized immune structure, with stromal cells exhibiting notable spatial heterogeneity that supports the formation of TCZ, BCZ, and MZ^8^. The TCZ is primarily composed of TRCs, which secrete IL-7, CCL19, and CCL21 to support T-cell survival and DCs recruitment^76^, ^77^. Within BCZ, FDCs and CXCL13-expressing BRCs promote B-cell activation and GC reactions^8^, ^78^. T-B border reticular cells (TBRCs), located at the TCZ-BCZ interface, express Gremlin-1 and contribute to dendritic cell homeostasis^79^. Marginal reticular cells (MRCs), characterized by MadCAM-1 and Ch25h expression, are localized beneath the marginal sinus and facilitate immune cell migration between the MZ and WP^8^. In addition, MRCs sustain the function of CD169⁺ marginal sinus macrophages through the RANKL pathway, thereby limiting blood-borne antigen dissemination and maintaining tolerance to self-antigens^80^. MRCs are distinct from bridging channel reticular cells (BCRCs), which form the structural framework of the BCs and provide a supportive niche for SIRPα⁺ cDC2s^38^, ^46^. BCRCs also secrete the chemokine CCL21, recruiting both naive and activated lymphocytes and guiding their transit across the MZ into the RP for blood-borne immune surveillance^44^. Together, these heterogeneous stromal cells form a structural and signaling platform essential for adaptive immune responses.

In the RP, heterogeneous RPRCs and specialized sinusoidal endothelial cells create a niche for iron recycling, blood filtration and EMH^8^, ^81^-^83^. RPRCs establish an extensive meshwork throughout the splenic cords and are characterized by the expression of Tcf21, Wt1, CXCL12, and stem cell factor. These cells play critical roles in maintaining plasma cell localization and sustaining hematopoietic activity^82^, ^84^. They also secrete colony-stimulating factor-1 (CSF-1) to sustain iron-handling RPMs^82^. Sinusoidal endothelial cells facilitate the clearance of senescent erythrocytes and pathogen surveillance, forming an efficient immune filtration system in the RP^75^, ^85^.

The splenic vasculature enters through the hilum and extends into both the WP and RP. It is accompanied by stromal cells that comprise the perivascular stromal niche (Figure 2C)^86^. α-SMA⁺ smooth muscle-like mural cells around CAs regulate vascular tone and perfusion^87^. CD34⁺ adventitial reticular cells located around CAs contribute to vascular morphogenesis and stability^88^. The splenic nerve runs along the CAs and extends into the TCZ, where it is supported by podoplanin-expressing glial cells^86^. Additionally, a THY1⁺ TRCs subset located around CAs produces vascular regulatory factors and may direct T cell entry into the WP^8^. These THY1⁺ TRCs, together with CXCL9⁺ TRCs, contribute to the architecture of the TCZ^86^. Collectively, these perivascular stromal cells regulate blood flow, immune cell trafficking, and tissue homeostasis, forming a crucial interface between the circulatory and immune systems.

### 3.3 Neural Innervation

**Figure 4:**
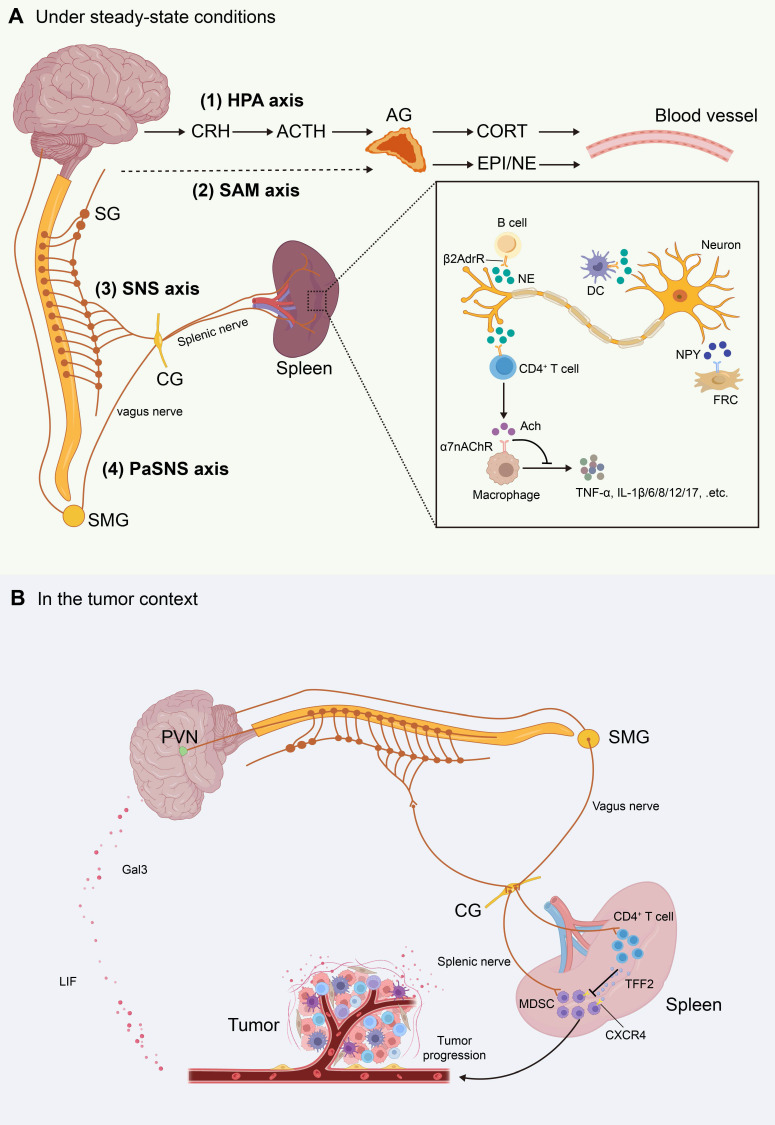
** Schematic illustration of the splenic neural network and neuroimmune crosstalk.** This figure summarizes the anatomic organization of major neuroendocrine and autonomic pathways that regulate the spleen, and their roles in neuroimmune communication. **(A)** Under steady-state conditions. The splenic neural network is depicted as four major circuits: (1) the hypothalamic-pituitary-adrenal (HPA) axis, (2) the sympathetic-adrenal-medullary (SAM) axis, (3) the sympathetic nervous system (SNS), and (4) the parasympathetic nervous system (PaSNS). The inset illustrates neuroimmune signaling within the spleen. Sympathetic splenic nerve fibers form varicosities near immune cells and release norepinephrine (NE). NE binds β2-adrenergic receptors (β2AdrR) on CD4⁺ T cells, B cells, and dendritic cells (DCs). Activated CD4⁺ T cells produce acetylcholine (Ach), which signals through α7 nicotinic acetylcholine receptors (α7nAChR) on macrophages. This pathway suppresses proinflammatory cytokine release. Neuropeptide Y (NPY) released from splenic nerves also modulates immune responses and may regulate fibroblastic reticular cell (FRC) function via its receptors. **(B)** In the tumor context. Tumor-brain-spleen signaling can be rewired. Across several tumor models, tumor-derived leukemia inhibitory factor (LIF) and galectin-3 (Gal3) activate brain pathways and enhance splenic sympathetic outflow. This promotes expansion of myeloid-derived suppressor cells (MDSCs) and upregulates their immunosuppressive programs. Pharmacologic or genetic blockade of tumor-derived LIF or Gal3 attenuates brain responses and restrains tumor progression. In contrast, vagus nerve signaling can engage splenic memory CD4⁺ T cells to release the anti-inflammatory peptide trefoil factor 2 (TFF2). TFF2 suppresses MDSC expansion via CXCR4-dependent signaling. This vagal protective axis is impaired in colorectal cancer. **Abbreviations:** CRH, corticotropin-releasing hormone; ACTH, adrenocorticotropic hormone; CORT, corticosterone; EPI, epinephrine; NE, norepinephrine; Ach, acetylcholine; HPA, hypothalamic-pituitary-adrenal; SAM, sympathetic-adrenal-medullary; SNS, sympathetic nervous system; PaSNS, parasympathetic nervous system; DC, dendritic cell; β2AdrR, β2-adrenergic receptor; α7nAChR, α7 nicotinic acetylcholine receptor; NPY, neuropeptide Y; FRC, fibroblastic reticular cell; CG, celiac ganglion; SG, sympathetic ganglion; SMG, superior mesenteric ganglion; PVN, paraventricular nucleus; LIF, leukemia inhibitory factor; Gal3, galectin-3; MDSC, myeloid-derived suppressor cell; TFF2, trefoil factor 2.

The nervous and immune systems are highly interconnected, with the “brain-spleen” neural axis serving as a crucial pathway for central regulation of peripheral immune responses. Studies have shown that central autonomic centers such as the hypothalamus and amygdala can transmit sympathetic signals to the spleen via the celiac and superior mesenteric ganglia, thereby establishing an inter-organ neuroimmune network^89^, ^90^. These sympathetic axons, known collectively as the splenic nerve, follow the splenic arterioles into the parenchyma, where they arborize densely around the CAs of the WP and extend into TCZ and BCZ (Figure 4)^91^. Electron microscopy and tissue-clearing studies have confirmed that these NE-releasing fibers form “bead-like” synapse-like terminals on the surfaces of T cells, B cells, and DCs. These terminals create localized microdomains for rapid neurotransmitter release and β2-adrenergic receptor (β2AdrR)-mediated immune sensing^8^. Neuropeptide Y (NPY) is another key neurotransmitter released by splenic nerves, modulating immune responses and FRCs function via its receptors^9^. Notably, neural density is significantly higher in TCZ compared to BCZ, suggesting spatial stratification of sympathetic regulation^92^.

Although direct projections of parasympathetic or vagal nerve terminals into the splenic parenchyma remain unproven, evidence suggests that the vagus nerve can modulate splenic nerve activity indirectly by influencing sympathetic output from the celiac ganglion^93^, ^94^. Vagal stimulation increases acetylcholine (ACh) levels in the spleen, which acts on α7 nicotinic acetylcholine receptors (α7nAChR) expressed on macrophages to suppress proinflammatory cytokines such as tumor necrosis factor-α (TNF-α) and IL-6, constituting the cholinergic anti-inflammatory reflex^94^. Some studies also propose the existence of a “C1 neuron-sympathetic-splenic nerve-spleen-kidney” multilevel regulatory axis, further emphasizing the complexity of splenic neuroimmune interactions^95^.

Splenic nerve activity is further governed by the hypothalamic-pituitary-adrenal (HPA) and sympathetic-adrenal-medullary (SAM) axes (Figure 4)^96^. In the HPA axis, neurons in the hypothalamus release corticotropin-releasing hormone (CRH), which targets the anterior pituitary, prompting the secretion of adrenocorticotropic hormone (ACTH). ACTH then induces the adrenal cortex to secrete corticosterone (CORT) into the bloodstream, which increases the firing frequency of the splenic nerve^97^. Concurrently, the SAM axis promotes adrenal medullary secretion of epinephrine (EPI) and norepinephrine (NE), activating adrenergic receptors in spleen and amplifying sympathetic-immune signaling^36^. Thus, splenic neuroimmune regulation operates not only within localized synaptic microenvironments but is also embedded in the broader stress-endocrine network, facilitating spatiotemporal integration of immune responses.

## 4. Tumor-Induced Rewiring of Splenic Niches

**Figure 5:**
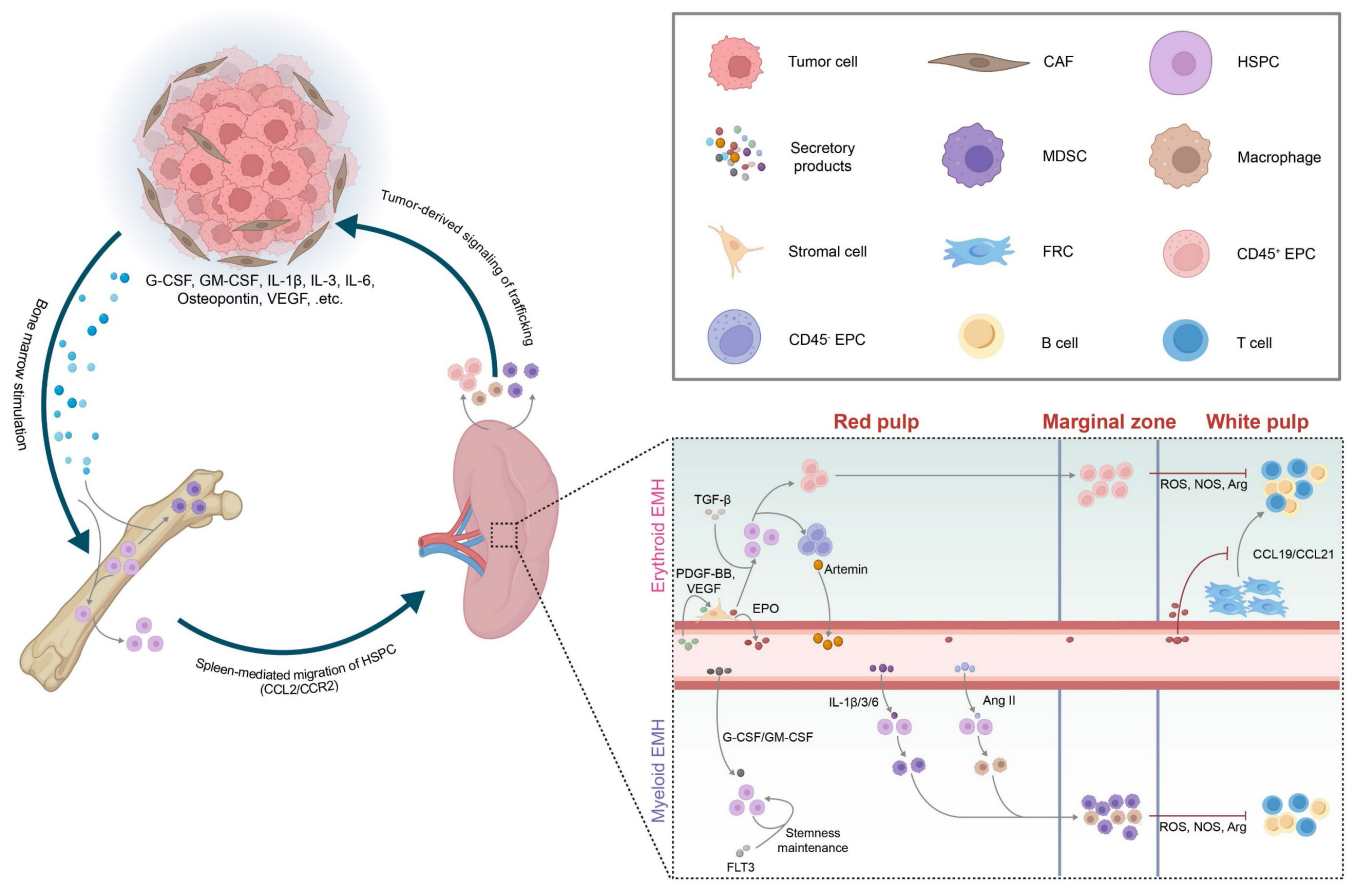
** Tumor-induced rewiring of splenic niches.** Tumor-derived secretory factors activate myelopoiesis in the bone marrow and promote the migration of hematopoietic stem and progenitor cells (HSPCs) to the spleen, where they initiate extramedullary hematopoiesis (EMH). The inset highlights erythroid EMH (upper) and myeloid EMH (lower) across the red pulp, marginal zone, and white pulp, together with representative niche cues. Myeloid-derived suppressor cells (MDSCs), tumor-associated macrophages (TAMs), and erythroid progenitor cells (EPCs) generated through splenic EMH contribute to local immunosuppression within the spleen. These cells can also be mobilized to primary tumors or metastatic sites, reinforcing a feed-forward loop that further accelerates tumor progression. This schematic is based primarily on insights from mouse models. **Abbreviations:** CAF, cancer-associated fibroblast; HSPC, hematopoietic stem and progenitor cell; EMH, extramedullary hematopoiesis; MDSC, myeloid-derived suppressor cell; TAM, tumor-associated macrophage; EPC, erythroid progenitor cell; FRC, fibroblastic reticular cell; G-CSF, granulocyte colony-stimulating factor; GM-CSF, granulocyte-macrophage colony-stimulating factor; IL, interleukin; Ang II, angiotensin II; VEGF, vascular endothelial growth factor; TGF-β, transforming growth factor-β; EPO, erythropoietin; PDGF-BB, platelet-derived growth factor-BB; FLT3, Fms-like tyrosine kinase 3; ROS, reactive oxygen species; NOS, nitric oxide synthase; Arg, arginase; CCL2, CC chemokine ligand 2; CCR2, CC chemokine receptor 2; CCL19/CCL21, CC chemokine ligand 19/21; ARTN, artemin.

Tumors are systemic diseases whose progression not only shapes the local tumor microenvironment (TME) but also systemically rewires distant immune-hematopoietic organs such as the spleen. This systemic modulation occurs through tumor-derived cytokines, extracellular vesicles, and activation of neuroendocrine stress axes. This section outlines tumor-induced splenic EMH, bidirectional tumor-spleen cellular trafficking, and the systemic signals mediating these changes (Figure 5).

### 4.1 Extramedullary Hematopoiesis

Tumor-induced splenic EMH is commonly observed across various murine solid tumor models, including melanoma^98^, lung adenocarcinoma^99^, HCC^5^, and breast cancer^100^, ^101^, as well as in patients with advanced solid tumors^11^, ^102^. It is characterized by splenomegaly, increased spleen index, and repopulation of the splenic RP by HSPCs, followed by robust differentiation^2^. Aberrant HSPCs recruitment underlies splenic EMH and is regulated by the CCL2-CCR2 axis, as well as by gain-of-function mutations in the LNK gene that enhance HSPC self-renewal^5^, ^103^, ^104^.

In tumor-bearing hosts, EMH is predominantly characterized by a myeloid-biased differentiation of HSPCs, largely driven by tumor-derived cytokines^11^. This skewed hematopoiesis leads to the substantial expansion of tumor-associated macrophages (TAMs) and MDSCs^105^. Based on morphology and surface phenotype, MDSCs are classified as monocytic (M-MDSC) and polymorphonuclear (PMN-MDSC) subtypes^106^. M-MDSCs are characterized in mice as CD11b⁺Ly6C^hi^Ly6G⁻ and in humans as CD11b⁺CD14⁺HLA-DR⁻/^lo^CD15⁻; PMN-MDSCs are defined in mice as CD11b⁺Ly6C^lo^Ly6G⁺ and in humans as CD11b⁺CD14⁻CD15⁺^106^. These MDSCs accumulate in the splenic MZ, where they closely interact with periarteriolar T cells. Through the production of reactive oxygen species (ROS) and the activity of immunosuppressive enzymes such as nitric oxide synthase (NOS) and arginase (Arg), they facilitate antigen-specific CD8⁺ T cell tolerance (Figure 5)^37^, ^103^. Tumor-infiltrating MDSCs additionally employ NO and Arg1 to reinforce local immunosuppression^107^-^110^.

Besides myeloid bias, the hypoxic TME and tumor-induced anemia can further drive erythroid-biased EMH^1^, ^5^, ^11^. The spleen accumulates abundant erythroid progenitor cells (EPCs), which exist in two subpopulations: CD45⁺ and CD45⁻ EPCs, both expressing Ter119⁺/CD71⁺^111^, ^112^. These EPCs are markedly expanded in lung adenocarcinoma and melanoma models and regress following tumor resection, indicating their persistent induction by the TME^112^. CD45⁺ EPCs release ROS that disrupt peripheral CD8⁺ T cell activation^111^, whereas CD45⁻ EPCs secrete the neurotrophic factor artemin, which accelerates tumor growth and correlates with poor prognosis in HCC patients (Figure 5)^112^. Notably, ERK1-deficient mice exhibit selective enhancement of erythroid-biased EMH without affecting myeloid expansion, suggesting a lineage-specific regulatory role of the ERK1-BMP4 pathway^113^.

### 4.2 Bidirectional Cell Trafficking

Beyond acting as a site of EMH that generates immunosuppressive cells, the spleen maintains dynamic bidirectional communication with tumors through a complex chemotactic network. On one hand, the spleen serves as a CCR2-dependent reservoir of monocytes, which are massively recruited to primary tumor sites and differentiate into TAMs under the influence of hypoxia and tumor-derived lactate in TME^99^, ^105^. On the other hand, tumors can redirect peripheral cells to the spleen to reshape distant immunity. In tumor-bearing mice, low levels of prostaglandin E2 (PGE2) have been shown to promote the recruitment of T cells from the thymus to the spleen. These splenic T cells subsequently accelerate tumor growth, although the underlying mechanisms remain poorly understood^114^. Similarly, breast cancer has been reported to drive neutrophil accumulation in the spleen. In a study by Wang *et al.*^115^, splenic stromal cells produced the chemokine CCL9, which mobilized neutrophils from the bone marrow into the spleen. These neutrophils aggregated in the WP, forming a glucose-depleted microenvironment that rendered local T cells metabolically paralyzed and weakened systemic antitumor responses. Through this reciprocal trafficking route, tumors and the spleen establish a dynamic “supply-demand” circuit that continuously supports tumor growth and metastasis.

### 4.3 Tumor-Derived Systemic Signals

In the context of myeloid-bias EMH signals, tumor-secreted granulocyte colony-stimulating factor (G-CSF) and granulocyte-macrophage colony-stimulating factor (GM-CSF) not only drive HSPCs toward MDSCs within the bone marrow, but also promote peripheral mobilization and splenic EMH (Figure 5)^116^-^118^. First, G-CSF induces proteases that cleave CXCL12-CXCR4 retention signals^119^, ^120^. Then, vascular endothelial growth factor (VEGF) binding to VEGFR2 dilates bone marrow microvessels^121^. Together, these changes facilitate the egress of HSPCs into the peripheral circulation. Mobilized HSPCs subsequently enter the spleen via the CCL2-CCR2 axis, with CCL2 mainly produced by splenic VE-cadherin⁺ endothelial cells^103^ and nestin⁺ reticular cells^5^. Tumor-derived cytokines such as IL-6, IL-3, and IL-1β further steer HSPCs toward M-MDSC and PMN-MDSC differentiation^5^, ^37^, a skewing that can be reversed by tumor resection or blockade of IL-1 or G-CSF^101^. Osteopontin and bone morphogenetic protein-4 (BMP4) exert opposing effects on granulopoiesis—osteopontin promotes PMN-MDSC output, whereas BMP4 suppresses it^122^, ^123^. Synergistic stimulation of HSPCs by G-CSF, GM-CSF, and Flt3 ligand (FLT3L) induces Hox gene upregulation, thereby sustaining HSPCs in an undifferentiated, proliferative state and establishing a splenic niche that supports persistent EMH and ongoing MDSC production^124^. Additionally, HSPCs are skewed toward TAM differentiation by tumor-derived angiotensin II (Ang II)^125^.

Regarding erythroid-biased EMH signals, platelet-derived growth factor-BB (PDGF-BB) and VEGF activate splenic PDGFR-β⁺ stromal cells to secrete erythropoietin (EPO). EPO, together with TGF-β/Smad3 signaling, expands the CD45⁻ Ter119⁺/CD71⁺ EPC pool (Figure 5)^112^, ^126^, ^127^. This process also suppresses CCL19 and CCL21 production by TRCs, thereby impeding T cell homing within the spleen^128^. Meanwhile, VEGF-VEGFR2 signaling can directly impair T and B cell development in the spleen, further dampening adaptive immunity^129^.

In terms of trafficking cues, tumor cells and cancer-associated fibroblasts (CAFs) produce CCL2 and a range of CXC chemokines that recruit splenic MDSCs and other EMH-derived myeloid cells to the tumor^105^, ^130^, ^131^. Additionally, CXCL5 and CXCL7 released by circulating tumor cells promote PMN-MDSCs dissemination to peripheral blood^132^. Collectively, these tumor-derived signals sustain the tumor-spleen feedback loop and establish a systemic immunosuppressive environment conducive to tumor progression.

### 4.4 Convergent Regulation of Splenic EMH and Cell Trafficking

Myeloid- and erythroid-biased EMH are often viewed as parallel processes. Direct evidence for reciprocal “shaping” between EPCs and MDSC/TAM populations remains limited. Some studies report that tumor-associated EPCs upregulate immunoregulatory cytokines such as IL-10 and TGF-β^6^. These mediators are known to sustain suppressive programs in MDSCs and to promote M2-like polarization of TAMs^133^, ^134^. In most experimental settings, however, the dominant sources of IL-10 and TGF-β are myeloid cells themselves and tumor tissues. Whether EPC-derived cytokines meaningfully contribute to maintaining MDSC/TAM phenotypes in the spleen or within tumors therefore remains an open question. By contrast, tumor-derived systemic signals provide clearer links between EMH and cell trafficking. In murine settings, G-CSF mobilizes HSPCs from the bone marrow to the spleen and helps maintain HSPC stemness, thereby supporting splenic EMH^124^. VEGF promotes erythroid-biased EMH through the EPO-EPC axis. In parallel, VEGF can also facilitate mobilization of myeloid progenitors and their recruitment to pre-metastatic sites^135^. TGF-β further connects these processes by enhancing EPC output and, meanwhile, promoting the accumulation of TAMs within the pre-metastatic niche^136^. Taken together, current evidence more strongly supports convergence at the level of shared upstream signals. It also highlights a major knowledge gap in myeloid-erythroid crosstalk.

## 5. Splenic Neuroimmune Crosstalk

Building on the cytokine-driven and trafficking-mediated changes described above, tumors can also engage neuroendocrine circuits to modulate splenic output. The spleen sits at a key neuroimmune interface and integrates autonomic and endocrine inputs to regulate homeostasis, systemic inflammation, and antitumor immunity^137^, ^138^. Under steady-state conditions, sympathetic nerve activation influences the trafficking of immune cells in circulation and within tissues^139^. For example, circadian rhythm-driven fluctuations in sympathetic activity determine peaks and troughs in NE secretion; NE promotes lymphocytes entry into lymph nodes and inhibits their egress^140^, ^141^. Sympathetic overactivation also induces vasoconstriction, which limits oxygen supply and further impacts lymphocytes migration^142^. In endotoxemia or ischemia-reperfusion models, vagus nerve stimulation activates splenic sympathetic nerves via the celiac ganglion, increasing ACh levels in splenic tissue^143^-^145^. ACh binding to α7nAChR on macrophages significantly suppresses TNF-α and IL-6 expression, thereby limiting systemic inflammatory fluctuations. Alterations in the cholinergic-sympathetic axis have been implicated in regulating various immune processes, including antibody generation by B cells during pneumococcal infection, T cell activation under hypertensive conditions, and the mobilization of immune cells^146^.

In the tumor context, neuroimmune interactions have been increasingly reported. For instance, the presence of sympathetic or parasympathetic fibers within prostate tumors correlates with reduced patient survival^147^. Conversely, epidemiological studies indicate that men with spinal cord injuries may lose sympathetic or parasympathetic efferent function. These men exhibit a lower incidence of prostate cancer^148^. Such clinical observations of nervous system involvement in cancer initiation and progression have been substantiated by mouse models, providing critical mechanistic insights. Notably, recent studies suggest that cancer cells may actively exploit neural circuits to facilitate their survival. In a murine model of pancreatic ductal adenocarcinoma (PDAC), tumor cells secreted nerve growth factor (NGF), which promoted sympathetic axonal ingrowth and accelerated tumor progression^149^. Similarly, multiple human cancer cell lines were found to secrete pro-brain-derived neurotrophic factor (proBDNF), thereby enhancing tumor innervation^150^. Moreover, in murine models including PDAC, colon adenocarcinoma, and Apc^min/+^ intestinal tumors, peripheral cancers activated catecholaminergic neurons in the ventrolateral medulla, which in turn suppressed CD8⁺ T cell activity and promoted tumor growth^151^. Xu and colleagues further demonstrated across several tumor models that cancer cells release leukemia inhibitory factor (LIF) and galectin-3 (Gal3) to activate the brain, which subsequently drives splenic sympathetic activation, leading to expansion of MDSCs and upregulation of their immunosuppressive molecules^152^. Pharmacological or genetic blockade of tumor-derived LIF or Gal3 abrogated brain responses and significantly inhibited tumor progression. In contrast, vagus nerve activation has been shown to modulate splenic memory T cells to release the anti-inflammatory peptide TFF2, which suppresses MDSC expansion via CXCR4 signaling^153^. This protective mechanism, however, is impaired in the setting of colorectal cancer. Collectively, these findings highlight the complex and dynamic interplay between the nervous system and tumors, particularly the splenic neuroimmune crosstalk in the tumor context, which warrants further in-depth investigation.

## 6. Therapeutic Strategies Targeting the Spleen

Cancer therapy not only reshapes the suppressive or activating pathways within the TME but also frequently relies on cooperation with the host immune system to enhance therapeutic efficacy^154^, ^155^. Emerging evidence highlights the spleen not only as a classical peripheral immune organ but also as a critical hub for regulating tumor-associated myelopoiesis, systemic immunosuppression, and chronic inflammation, making it a novel target for therapeutic intervention^11^. There are several advantages to targeting the spleen. First, its rich blood supply facilitates the delivery of drugs and cytokines. Second, the spleen is enriched with tumor-induced immunosuppressive cells (e.g., MDSCs and EPCs), and suppressing their generation or trafficking can relieve systemic immunosuppression. Third, the spleen is closely connected to the autonomic nervous system, offering neuromodulatory entry points.

**Figure 6:**
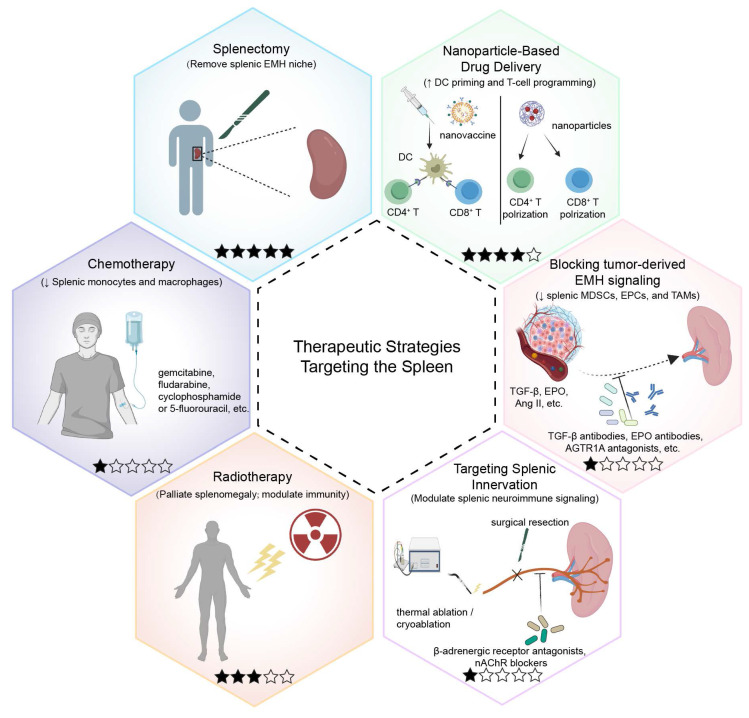
** Therapeutic strategies that directly or indirectly target the spleen in tumor.** The hexagonal schematic summarizes six current or emerging approaches designed to modulate splenic structure or function and thereby counter tumor-driven immune dysregulation. A five-star maturity scale is included for each strategy to visually summarize translational readiness, where each filled star indicates one level of evidence (1-5): 1, preclinical only; 2, preclinical plus case report/series; 3, retrospective clinical cohorts; 4, prospective studies/registered trials; and 5, randomized controlled trials (RCTs) and/or guideline-supported or widely adopted practice (indication-specific). Splenectomy (upper left) removes the spleen and eliminates a major reservoir of tumor-induced suppressive populations. Nanoparticle-based drug delivery (upper right) uses nanovaccines or drug-loaded nanoparticles to prime splenic antigen-presenting cells (APCs), particularly dendritic cells (DCs), and to program or reprogram T-cell responses. Blocking tumor-derived EMH signaling (middle right) targets mediators such as transforming growth factor-β (TGF-β), erythropoietin (EPO), and angiotensin II (Ang II) that drive splenic extramedullary hematopoiesis (EMH). Representative interventions include TGF-β neutralizing antibodies, anti-EPO antibodies, and angiotensin II receptor type 1A (AGTR1A) antagonists. Targeting splenic innervation (lower right) aims to modulate splenic neuroimmune signaling, for example by β-adrenergic receptor antagonists (e.g., propranolol), nicotinic acetylcholine receptor (nAChR) blockade, or denervation approaches (surgical, thermal ablation, or cryoablation). Radiotherapy (lower left) delivers focal splenic irradiation, primarily used clinically for palliation, and may modulate immunity. Chemotherapy (middle left) includes cytotoxic agents (e.g., gemcitabine, fludarabine, cyclophosphamide, or 5-fluorouracil) that can decrease splenic immunosuppressive cell output. **Abbreviations**: AGTR1A, angiotensin II receptor type 1A; Ang II, angiotensin II; APC, antigen-presenting cell; DC, dendritic cell; EMH, extramedullary hematopoiesis; EPO, erythropoietin; nAChR, nicotinic acetylcholine receptor; TGF-β, transforming growth factor-β; RCTs, randomized controlled trials.

In this section, we discuss different therapeutic strategies targeting the spleen (Figure 6). We classify each approach by tumor setting, mechanistic target within the tumor-spleen axis, and strength of evidence. Table 1 summarizes the key mechanism, evidence level, and outcomes, while the text below distinguishes clinical data from preclinical findings and discusses major translational limitations.

### 6.1 Splenectomy

Splenectomy is discussed in oncology for two fundamentally different reasons that should be appraised separately. First, splenectomy may be performed as a technical component of oncologic surgery to enable complete cytoreduction or en bloc resection when the spleen/splenic hilum is involved. Second, splenectomy has been proposed as an immunomodulatory strategy, based on the concept that the spleen can serve as a reservoir for tumor-induced suppressive populations (e.g., MDSCs and EPCs) that propagate systemic immunosuppression^133^, ^156^ (Table 1).

#### 6.1.1 Preclinical evidence

In several murine models, splenectomy reduced splenic expansion of suppressive myeloid/erythroid populations and was accompanied by enhanced cytotoxic T-cell activity and tumor control^112^, ^157^, ^158^. However, these effects are context dependent and not uniformly favorable across experimental settings. In addition, the reported impact of splenectomy on immune composition at primary and metastatic sites has been inconsistent across models^158^-^161^. For instance, Sevmis *et al.*^161^ showed that splenectomy promoted the accumulation of circulating and tumor-infiltrating MDSCs with a transient deceleration of primary tumor growth but increased lung metastasis over the long term. Collectively, murine data support a mechanistic link between the spleen and systemic immunoregulation. However, heterogeneity in splenectomy timing, tumor model, and study design may contribute to inconsistent findings.

#### 6.1.2 Clinical evidence

In clinical practice, splenectomy is most commonly performed for technical/oncologic indications rather than for immune modulation. When gastric cancers or pancreatic tumors directly invade the spleen or involve the splenic hilum, concomitant splenectomy may be required to enable en bloc resection and achieve an R0 margin^162^, ^163^. However, for proximal advanced gastric cancer with clinically negative splenic hilar nodes, retrospective cohorts suggest that prophylactic splenectomy increases perioperative morbidity without improving long-term outcomes^164^-^166^. These findings are further supported by a multicenter randomized controlled trial (RCT) showing the noninferiority of spleen preservation in overall survival^167^. In cytoreductive surgery for advanced ovarian cancer, splenectomy is typically undertaken to achieve optimal cytoreduction when tumor involves the spleen or splenic hilum^168^. Splenectomy can be performed safely in selected patients but does not confer a survival advantage and may increase perioperative burden^169^.

Beyond technical/oncologic indications, splenectomy may also be considered in patients with cancer complicated by hypersplenism, where cytopenia can limit treatment delivery. In hematologic malignancies with symptomatic splenomegaly, splenectomy may provide symptomatic relief and hematologic improvement^170^. In the retrospective cohort study by Zhou *et al.*^171^, concomitant splenectomy was associated with improved disease-free survival (DFS) in patients with early HCC and cirrhosis-related hypersplenism undergoing hepatectomy. For patients with unresectable HCC complicated by cirrhosis-related hypersplenism, immune checkpoint inhibitors (ICIs) represent the standard first-line therapy^172^. However, cytopenia resulting from hypersplenism often hinders the consistent administration of ICIs. In such cases, preemptive splenectomy to correct hypersplenism may offer a viable opportunity for patients to receive subsequent ICIs therapy^173^. Nevertheless, whether splenectomy can further enhance the efficacy of ICIs remains to be validated in prospective studies.

#### 6.1.3 Translational challenges

Any putative oncologic benefit must be weighed against the well-established consequences of asplenia^174^-^177^. Splenectomy removes both suppressive reservoirs and protective splenic immune functions, while introducing infectious and thrombotic risks that may offset any potential oncologic benefit. Moreover, interpretation of immunomodulatory effects requires caution because human and murine spleens differ substantially in microanatomy and compartment organization. This may limit direct translation of murine splenectomy phenotypes to human immune remodeling.

### 6.2 Nanoparticle-Based Drug Delivery Systems

After intravenous injection, most nanoparticles are initially taken up by liver Kupffer cells and splenic phagocytic monocytes^178^. The spleen's unique blood-filtering architecture and slow blood flow create a natural “reservoir” for drug accumulation^179^, ^180^. Enriched in APCs, the spleen enables rationally designed spleen-targeted nanovaccines to be internalized by APCs and deliver antigens to T and B cells within specific splenic compartments, rapidly initiating and sustaining robust humoral and cellular immune responses^42^. In parallel, spleen-directed nanoparticles can directly activate CD4⁺ and CD8⁺ T cells^181^. Spleen-targeted nanoplatforms have been developed to deliver diverse cargos including mRNA, siRNA, plasmid DNA, and Cas9 mRNA/sgRNA or Cas9-protein complexes^182^, ^183^. To enhance targeting specificity, carriers are conjugated with ligands, antibodies, or protein coronas to direct delivery to splenic immune subpopulations 184 (Table 1).

#### 6.2.1 Preclinical evidence

In nanovaccine development, Shen *et al.*^185^ engineered a DEC-205 antibody-conjugated nanoparticle that selectively activated CD8α⁺ DCs, inducing potent cytotoxic T lymphocyte responses. Similarly, Kurosaki *et al.*^186^ developed a γ-PGA-coated DNA vaccine that selectively transfected cells in the splenic MZ and significantly inhibited melanoma growth and metastasis. α-mannose-functionalized poly(β-amino ester) nanoparticles have also been shown to target splenic APCs, enhancing mRNA vaccine efficiency^187^. Wu *et al.*^188^ reported a DNA-polymer nanoparticle platform conjugated with red blood cells to preferentially accumulate in the spleen, enhancing neoantigen expression by APCs and eliciting potent T cell responses to suppress HCC progression. When combined with ICIs, this vaccine can elicit a robust systemic immune response with long-term tumor-specific immunological memory. This combination can induce complete tumor regression and effectively prevent tumor recurrence and spontaneous lung metastasis. In a murine melanoma model, PD-1-blocking antibodies encapsulated in biodegradable polymeric nanoparticles were delivered to splenic APCs and elicited strong antitumor responses^181^.

Nanoparticles have also been used to directly modulate CD4⁺ and CD8⁺ T cells in the spleen. Pan *et al.*^189^ designed sLNPs-OVA/MPLA nanoparticles co-delivering mRNA antigen and a TLR4 agonist to the spleen, triggering synergistic immune activation and robust Th1 polarization. αCD3-targeted lipid nanoparticles (LNPs) promoted splenic CD8⁺ T cell accumulation, migration from WP to RP, and differentiation into memory and effector subsets 190. Nanotechnology also enables *in vivo* CAR-T cell engineering. Álvarez-Benedicto *et al.*^191^ utilized spleen selective organ targeted LNPs to deliver Cre recombinase mRNA and CAR-encoding mRNA to splenic T cells. This generated functional CAR-T cells in situ in a B cell lymphoma model, which reduced liver metastasis and prolonged survival. Similarly, Billingsley *et al.*^192^ designed ionizable LNPs for *in vivo* delivery of mRNA to splenic T cells for CAR-T reprogramming.

#### 6.2.2 Clinical evidence

Most spleen-directed nanoplatforms remain preclinical, with only a limited subset having entered early-phase clinical evaluation (Table 1). Kranz *et al.*^193^ developed intravenously administered RNA-lipoplexes (RNA-LPX), which were taken up by splenic DCs and induced robust antigen-specific T-cell responses. Notably, a phase I dose-escalation trial of RNA-LPX encoding shared tumor antigens was reported as ongoing, and the first three melanoma patients showed induction of IFN-α and antigen-specific T-cell responses. Most spleen-targeted nanoplatforms are administered systemically. They may therefore be applicable to unresectable or advanced cancers.

#### 6.2.3 Translational challenges

Despite encouraging results in early animal studies, spleen-targeted nanomedicines still face multiple critical barriers before they can be successfully translated into clinical practice. After intravenous administration, most nanocarriers are rapidly coated by a dynamically formed protein corona and cleared by the mononuclear phagocyte system, particularly hepatic Kupffer cells. In many studies, more than 70% of the injected dose ultimately accumulates in the liver, and only a small fraction actually reaches the TCZ and BCZ in the spleen^42^. Hepatic and renal clearance further redistribute nanoparticles according to their size, charge, and morphology, making it difficult to achieve sufficient splenic accumulation without excessively increasing off-target toxicity^179^, ^194^. Within the spleen itself, RPMs and resident cells in the MZ constitute an additional “barrier,” efficiently phagocytosing incoming nanoparticles and thereby limiting their penetration into the WP, where they would need to interact with DCs or lymphocytes.

Safety and pharmacokinetics pose further challenges. Cationic or strongly immunostimulatory lipid nanoparticles can activate the complement system, trigger cytokine release, and even cause infusion-related reactions—issues that are particularly relevant in cancer patients requiring repeated dosing. For many mRNA nanovaccines, the effective residence time in the spleen is relatively short, with a functional half-life often below 6 hours, which may necessitate higher doses or frequent administration to sustain adequate immune activation^195^. In addition, most spleen-targeted nanoplatforms have been optimized primarily in murine models, even though mice and humans differ markedly in splenic architecture, hemodynamics, and the spatial organization of immune cells. As a result, tissue distribution patterns and effective doses observed in mice cannot be directly extrapolated to humans.

Finally, these formulations are typically chemically complex, multi-component products, which creates additional hurdles for large-scale manufacturing, batch-to-batch quality control, and regulatory evaluation. Taken together, spleen-targeted nanomedicines as a whole remain at an early translational stage, and systematic pharmacokinetic, safety, and dose-finding studies in humans will be essential before they can realistically enter routine cancer therapy.

### 6.3 Chemotherapy

Preclinical studies suggest that conventional cytotoxic agents can enhance antitumor therapy not only by directly killing tumor cells but also by remodeling the tolerogenic milieu of the spleen (Table 1). In murine models of sarcoma and lung cancer, low-dose gemcitabine, fludarabine, cyclophosphamide or 5-fluorouracil markedly reduce splenic Ly6C^hi^ monocytes and restore the effector function of exhausted CD8⁺ cytotoxic T lymphocytes^103^. Trabectedin, a marine-derived DNA-binding agent, has also shown antitumor activity in tumor-bearing mice by selectively inducing apoptosis of monocytes and macrophages^196^.

Immature erythroid cells are generally more susceptible to chemotherapy than mature erythrocytes, which are relatively less affected by cytotoxic agents targeting proliferating cells^197^. Given that splenic EMH-derived EPCs represent an immature erythroid population, they are theoretically more susceptible to cytotoxic chemotherapy.

However, this possibility has not been directly demonstrated and requires dedicated *in vivo* validation. Importantly, many cytotoxic drugs concomitantly deplete lymphoid pools, shrink the spleen and disturb its microarchitecture^103^. Some agents may also cause severe hemolytic anemia and thrombocytopenia by impairing liver function or disrupting hematologic homeostasis^198^. Therefore, more precise spleen-targeted delivery strategies are needed to preserve the benefits of chemotherapy while minimizing systemic toxicity^199^.

### 6.4 Radiotherapy

Clinically, localized splenic irradiation is an established palliative option for symptomatic splenomegaly caused by hematological malignancies and related disorders^200^-^202^ (Table 1). The available evidence is predominantly derived from retrospective cohorts, focusing on symptom relief and hematologic improvement. Accordingly, its impact on systemic antitumor immunity and oncologic outcomes remains poorly defined. Mechanistic insights into splenic irradiation-induced immune modulation are therefore drawn largely from preclinical studies. In a murine melanoma model, Chen *et al.*^203^ found that splenic irradiation increased IL-1β production in the spleen, which upregulated CXCR3 expression on T cells and enhanced their migratory capacity. When combined with tumor irradiation, splenic irradiation increased T-cell infiltration into tumors and improved local tumor control.

Overall, prospective human studies with predefined immune monitoring and clinically relevant dose-fractionation are needed. These studies should define how splenic irradiation reshapes the splenic microenvironment. They should also clarify whether it can be safely leveraged as an immunomodulatory adjunct beyond palliation. In retrospective cohorts, higher splenic doses have been associated with an increased risk of severe lymphopenia^204^-^207^. This becomes particularly relevant when radiotherapy or systemic chemotherapy is combined with ICIs. Lymphopenia can compromise ICI delivery and efficacy. It may also reflect broader immune injury. Therefore, spleen-sparing planning and dose-volume constraints should be considered whenever feasible. Key priorities include defining dose-volume thresholds and sequencing strategies that preserve splenic immune competence in ICI-based regimens.

### 6.5 Targeting Tumor-Derived Signals Driving Splenic EMH

The TME is often characterized by high cellular density, hypoxia, and chronic inflammation^208^. These factors promote the secretion of various signaling molecules by tumors, which remotely drive splenic EMH, particularly the expansion of MDSCs and EPCs, thereby inducing systemic immunosuppression. Consequently, targeting and blocking tumor-derived signals has emerged as a key strategy for modulating splenic EMH and improving the host immune status. At present, the evidence supporting these approaches in cancer largely comes from preclinical studies, and direct human data for modulating splenic EMH remain limited (Table 1).

TGF-β has been identified as a central driver of EPC expansion. Neutralizing antibodies against TGF-β markedly reduce the abundance of splenic EPCs in HCC^112^. The TGF-β superfamily ligand trap ACE-536 effectively attenuates Smad2/3 signaling and alleviates ineffective erythropoiesis in murine models of myelodysplastic syndrome^209^. Additionally, the combination of TGF-β antibodies and ICIs has shown synergistic antitumor effects in murine models of HCC^210^. However, whether this combination also modulates EPC expansion and function remains to be determined. EPO promotes tumor-induced splenic EMH and drives HSPCs toward erythroid differentiation^126^, ^127^, ^211^. Neutralizing antibodies against EPO have been shown to reduce EPCs generation and delay tumor growth in melanoma^212^. Another promising target is Ang II, which contributes to the maintenance and expansion of splenic HSPCs^125^. Use of angiotensin II receptor type 1A (AGTR1A) antagonists (e.g., losartan) or angiotensin-converting enzyme inhibitors (e.g., enalapril) can effectively block tumor-driven myeloid cell mobilization and reduce the accumulation of TAMs in lung cancer^125^. In traditional Chinese medicine, the herbal formula Danggui Buxue Tang has been shown to alleviate abnormal EPCs accumulation by modulating transcription factors Pu.1 and Gata-1, thereby inhibiting tumor progression and enhancing antitumor immune responses in melanoma^213^.

Overall, targeting tumor-derived cues represents a rational route to suppress splenic EMH and relieve systemic immunosuppression. However, the current evidence is largely preclinical, and future studies should prioritize mechanistic validation and translational evaluation in clinically relevant settings.

### 6.6 Targeting Splenic Innervation

Current evidence for targeting splenic innervation is limited. Human data are mainly observational and not spleen-specific, whereas mechanistic support largely comes from preclinical models (Table 1). In observational studies, long-term use of β-adrenergic receptor antagonists has been associated with reduced metastasis and mortality in breast and ovarian cancer^214^-^216^. Similar associations with improved survival have also been reported in NSCLC patients receiving radiotherapy, chemotherapy, or immunotherapy^217^-^219^.

Mechanistic support comes primarily from animal studies. In murine breast cancer models, β-adrenergic receptor antagonists therapy reduced tumor-associated macrophage infiltration, thereby slowing tumor metastasis^220^. In an Ehrlich carcinoma model, pharmacologic blockade of multiple nAChR subtypes (α6, α3β2, α9α10, and α7) suppressed tumor growth and enhanced splenic cytotoxic activity^221^. Moreover, across several mouse tumor models, splenic sympathetic denervation (e.g., sympathectomy) reduced splenic MDSC expansion and partially restored antitumor immunity^152^.

Collectively, these findings suggest that targeting splenic neuroimmune signaling may help counteract tumor-induced immunosuppression. Candidate strategies include β-adrenergic receptor antagonists (e.g., propranolol), nAChR blockade, and splenic denervation (surgical resection, thermal ablation, or cryoablation). However, current evidence is largely preclinical or observational. Key uncertainties remain regarding causality, target engagement, safety, and whether these interventions translate into clinically meaningful tumor control in prospective trials.

## 7. Spleen as a Biomarker

Increasing evidence suggests that alterations in splenic morphology and metabolism may serve as noninvasive indicators for tumor burden and therapeutic response^222^, ^223^. In a cohort of patients with pancreatic ductal adenocarcinoma undergoing FOLFIRINOX treatment, baseline splenomegaly was associated with poorer overall survival^223^. Among patients with colorectal liver metastases receiving neoadjuvant chemotherapy, greater increases in spleen volume were significantly correlated with worse survival outcomes^224^. Fluctuations in splenic volume have also been documented in NSCLC patients undergoing with chemoradiotherapy, although their prognostic or predictive value remains to be fully elucidated^225^. Baseline spleen volume and its longitudinal changes during treatment have been explored as potential surrogate indicators of immunotherapy response in advanced urothelial carcinoma^226^, HCC^227^, and NSCLC^228^.

On the other hand, tumors can mediate splenic metabolic reprogramming, notably characterized by enhanced glycolytic activity in the spleen^115^. Increased glucose metabolism in the spleen, assessed using ¹⁸F-fluorodeoxyglucose (¹⁸F-FDG) positron emission tomography (PET), has been linked to unfavorable clinical outcomes in several tumors. These include breast cancer^229^, cervical cancer^230^, cholangiocarcinoma^231^, gastric cancer^232^, and colorectal cancer^233^. The mechanism may involve the recruitment of neutrophils that accumulate in the WP, deplete local glucose, and induce T cell dysfunction^115^. In a study of locally advanced cervical cancer, patients with higher splenic FDG uptake showed denser immune cell infiltration in the primary tumor but were more prone to disease progression and less likely to achieve pathologic complete response^234^. In metastatic melanoma, Seban *et al.*^235^ demonstrated that pre-treatment splenic total metabolic tumor volume and total lesion glycolysis were significantly associated with the response to immunotherapy. However, other studies have reported no clear association between splenic standardized uptake value and immunotherapy outcomes^236^, ^237^.

Importantly, many factors can influence splenic volume and ¹⁸F-FDG uptake. These include portal hypertension, infections, and hematologic disorders. Treatment-related adverse events can also change splenic morphology or metabolism independent of tumor-driven EMH. Therefore, current data on spleen-based imaging biomarkers should be interpreted as exploratory rather than definitive. Overall, splenic volume and metabolic activity are promising adjunctive imaging readouts. Nevertheless, prospective validation in well-defined multicenter cohorts with careful control of confounders is warranted to ensure their reproducibility, predictive accuracy, and generalizability. Standardized imaging protocols are also required before these measures can be incorporated into routine clinical decision-making.

## 8. Conclusion and Perspectives

The spleen has recently been re-recognized as a central regulator of tumor immunity. Tumors can rewire the splenic hematopoietic and stromal networks, shifting its microenvironment from immune defense to immune suppression, thereby promoting distant tumor growth and metastasis. The role of the spleen in tumor immune regulation is an intriguing topic, and increasing attention has been given to the spleen as a potential therapeutic target. In this study, we systematically reviewed several representative spleen-targeting strategies, including splenectomy, nanomedicine delivery, chemotherapy, radiotherapy, molecule inhibitors, and neurostimulation. These approaches comprehensively demonstrate the potential of targeting splenic immune cells, neural circuits, and EMH signaling in cancer therapy.

However, several challenges remain in the clinical translation of these therapeutic strategies. One major limitation lies in the anatomical and functional disparities between murine and human spleens, which may result in species-specific differences in antigen presentation and the initiation of adaptive immunity. Consequently, results derived from preclinical mouse models do not always translate into effective clinical outcomes. Moreover, the neuroimmune regulatory networks within the spleen remain only partially characterized, leaving key mechanistic gaps unresolved. The spleen also harbors a diverse array of immune cell subsets that respond dynamically to both systemic and local stimuli, contributing to patient-to-patient variability in therapeutic efficacy. In addition, current physical and drug-based modulation strategies often fall short in terms of precision and reproducibility, limiting their potential to support genuinely personalized treatment approaches.

To overcome these limitations, future efforts might focus on the following aspects. First, the integration of single-cell resolution techniques (e.g., single-cell RNA sequencing and spatial transcriptomics) with temporally controlled splenectomy models may help elucidate how tumor-driven alterations in splenic architecture and cellular communication influence primary tumor development. These models may also shed light on pre-metastatic niche formation, tumor cell seeding, and dormancy. Second, leveraging artificial intelligence modeling and multi-modal data integration could enhance patient stratification and individualized decision-making. Third, rational design of nanomaterials may allow the construction of spleen-targeted delivery systems with high loading efficiency and acceptable safety margins. Finally, comprehensive preclinical and regulatory evaluations of acute and chronic toxicities, long-term immunologic effects, and potential off-target consequences are essential to support clinical translation.

In conclusion, the spleen represents a pivotal hub in tumor immune regulation, with promising potential as both a biomarker and therapeutic target. Multidisciplinary collaboration involving immunology, neuroscience, materials science and computational biology may deepen our understanding of the spleen's spatiotemporal dynamics under tumor influence. This integrated approach may also accelerate the clinical translation of spleen-targeted immunotherapies and open new frontiers in cancer treatment.

## Figures and Tables

**Table 1:** Stratified comparison of spleen-targeted therapeutic strategies in tumor

Strategy	Tumor type	Treatment	Main mechanism	Evidence level	Outcomes	Reference
Splenectomy	HCC	Splenectomy	Depletes splenic EPCs	Preclinical	Reduced primary tumor growth	112
	NSCLC	Splenectomy	Depletes splenic MDSCs	Preclinical	Reduced primary tumor growth	157
	Breast cancer	Splenectomy	Reduces accumulation of TAMs, TANs, and TIDCs in metastatic sites	Preclinical	No effect on primary tumor growth; reduced lung metastasis	158
	Breast cancer	Splenectomy	Promotes accumulation of circulating and tumor-infiltrating MDSCs	Preclinical	Transiently reduced primary tumor growth; increased lung metastasis	161
	Advanced ovarian cancer	Splenectomy vs no splenectomy	Enables optimal cytoreduction	Retrospective cohort	No significant survival benefit; longer length of stay; delayed initiation of chemotherapy	169
	Proximal advanced gastric cancer	Gastrectomy + splenectomy vs gastrectomy	Prophylactic splenectomy to enable splenic hilar lymph node dissection	Retrospective cohort	No significant survival benefit; increased postoperative morbidity	164-166
	Proximal advanced gastric cancer	Gastrectomy + splenectomy vs gastrectomy	Prophylactic splenectomy to enable splenic hilar lymph node dissection	RCT	No significant survival benefit; increased postoperative morbidity	167
	Advanced hematological malignancies	Splenectomy	Palliates splenomegaly	Retrospective cohort	Provided durable palliation for massive splenomegaly	170
	Early HCC with hypersplenism	Hepatectomy + splenectomy vs hepatectomy	Corrects cytopenia; reduces bleeding risk	Retrospective cohort	Improved DFS	171
	Unresectable HCC with hypersplenism	Splenectomy + targeted therapy + ICIs	Corrects cytopenia; reduces bleeding risk	Case report	Enabled systemic therapy; prolonged survival	173
Nanoparticle-based drug delivery systems	Melanoma	Trifunctional nanoparticle	Targets and activates CD8^+^ DCs	Preclinical	Reduced primary tumor growth	185
	Melanoma	γ-PGA-DNA vaccine	Achieves efficient gene transfection in splenic compartments	Preclinical	Reduced primary tumor growth; reduced metastasis	186
	HCC	RBC-driven neoantigen DNA vaccine	Preferentially accumulates in the spleen to promote the neoantigen expression by APCs	Preclinical	Reduced primary tumor growth	188
	Melanoma	anti-PD-1 nanoparticles	Promotes splenic DC uptake, maturation and subsequent T-cell priming	Preclinical	Reduced primary tumor growth	181
	Melanoma; lymphoma	mRNA and TLR4 agonist-LNPs	Drives spleen-enriched mRNA expression and Th1-skewed immune activation	Preclinical	Reduced primary tumor growth; reduced metastasis	189
	Breast cancer	aCD3-LNPs	Targets splenic T cells to induce activation, trafficking, and phenotypic reprogramming	Preclinical	Reduced primary tumor growth	190
	Melanoma	RNA-lipoplexes vaccines	Triggers IFNα release by plasmacytoid DCs and macrophages	Phase I trial	Induced type I IFN and antigen-specific T-cell responses	193
	Lymphoreplete B cell lymphoma	Spleen SORT LNPs	Enables in situ CAR-T cell generation	Preclinical	Reduced primary tumor growth; reduced metastasis	191
Chemotherapy	Sarcoma, lung cancer	Gemcitabine; fludarabine; cyclophosphamide; 5-fluorouracil	Depletes splenic Ly6C^hi^ monocytes and restores CTL function	Preclinical	Enhanced ACT efficacy; reduced tumor growth	103
	Fibrosarcoma; lung cancer; ovarian cancer	Trabectedin	Selectively depletes monocytes/macrophages in blood, spleen, and tumors	Preclinical	Reduced primary tumor growth	196
Radiotherapy	Hematologic malignancies and disorders	Splenic irradiation	Palliative splenomegaly	Retrospective cohort	Provided symptomatic relief and improved hematologic parameters	201, 202
	Melanoma	Splenic irradiation + tumor irradiation	Promotes T cell infiltration in the tumor microenvironment	Preclinical	Reduced primary tumor growth	203
Targeting tumor-derived signals driving splenic EMH	HCC	TGF-β antibody	Neutralizes TGF-β signaling	Preclinical	Reduced splenic EPC expansion	112
	Melanoma	EPO antibody	Neutralizes EPO signaling	Preclinical	Reduced EPC generation; reduced tumor growth	212
	Lung cancer	AGTR1A antagonist (losartan); ACEI (enalapril)	Inhibits Ang II signaling	Preclinical	Reduced TAM accumulation; reduced primary tumor growth	125
	Melanoma	Danggui Buxue Tang	Modulates erythroid-lineage transcriptional programs (Pu.1/Gata-1)	Preclinical	Reduced EPC accumulation; reduced primary tumor growth and metastasis	213
Targeting splenic innervation	Breast cancer	Propranolol	Blocks β-adrenergic signaling	Preclinical	Reduced TAM infiltration; reduced metastasis	220
	Ehrlich carcinoma	nAChR blockers	Blocks nAChR subtypes (α6, α3β2, α9α10, α7)	Preclinical	Reduced primary tumor growth	221
	Lung cancer; prostate cancer; colon cancer; breast cancer	Sympathetic ablation; sympathectomy	Removes splenic sympathetic inputs	Preclinical	Reduced splenic MDSCs; reduced primary tumor growth	152

**Abbreviations:** ACEI, angiotensin converting enzyme inhibitor; ACT, adoptive cell therapy; AGTR1A, angiotensin II receptor type 1A; Ang II, angiotensin II; APCs, antigen-presenting cells; CAR-T, chimeric antigen receptor T cells; CCR2, C-C chemokine receptor 2; CTL, cytotoxic T lymphocyte; DCs, dendritic cells; DFS, disease-free survival; EPCs, erythroid progenitor cells; EPO, erythropoietin; HCC, hepatocellular carcinoma; ICIs, immune checkpoint inhibitors; IFNα, interferon-α; LNPs, lipid nanoparticles; mRNA, messenger RNA; MDSCs, myeloid-derived suppressor cells; nAChR, nicotinic acetylcholine receptor; NSCLC, non-small cell lung cancer; PDAC, pancreatic ductal adenocarcinoma; RCT, randomized controlled trial; siRNA, small interfering RNA; SORT, spleen selective organ targeted; TAMs, tumor-associated macrophages; TANs, tumor-associated neutrophils; TIDCs, tumor-infiltrating dendritic cells; TLR4, toll-like receptor 4.
